# Can language representation models think in bets?

**DOI:** 10.1098/rsos.221585

**Published:** 2023-03-29

**Authors:** Zhisheng Tang, Mayank Kejriwal

**Affiliations:** Information Sciences Institute, USC Viterbi School of Engineering, 4676 Admiralty Way 1001, Marina Del Rey, CA 90292 USA

**Keywords:** language representation models, decision-making problems, preference elicitation, transformer neural networks, neural language models, cognitive science

## Abstract

In recent years, transformer-based language representation models (LRMs) have achieved state-of-the-art results on difficult natural language understanding problems, such as question answering and text summarization. As these models are integrated into real-world applications, evaluating their ability to make rational decisions is an important research agenda, with practical ramifications. This article investigates LRMs’ rational decision-making ability through a carefully designed set of decision-making benchmarks and experiments. Inspired by classic work in cognitive science, we model the decision-making problem as a bet. We then investigate an LRM’s ability to choose outcomes that have optimal, or at minimum, positive expected gain. Through a robust body of experiments on four established LRMs, we show that a model is able to ‘think in bets’ if it is first fine-tuned on bet questions with an identical structure. Modifying the bet question’s structure, while still retaining its fundamental characteristics, decreases an LRM’s performance by more than 25%, on average, although absolute performance remains well above random. LRMs are also found to be more rational when selecting outcomes with non-negative expected gain, rather than optimal or strictly positive expected gain. Our results suggest that LRMs could potentially be applied to tasks that rely on cognitive decision-making skills, but that more research is necessary before these models can robustly make rational decisions.

## 1. Introduction

Transformer neural network-based language representation models (LRMs), such as the bidirectional encoder representations from transformers (BERT) [1] and the generative pre-trained transformer (GPT) series of models [2,3], have led to impressive advances in natural language understanding. These models have significantly advanced state-of-the-art performance on a variety of natural language tasks, ranging from information extraction [4] and semantic role labelling [5], to text summarization [6], cross-lingual and multi-lingual understanding [7], and question answering [8]. Variants of such models [9–12] currently underlie the most successful systems on competition leaderboards hosted by the Allen Institute for Artificial Intelligence for several important benchmarks [13,14]. Domain-specific versions of these models have also achieved impressive performance in their respective domains (e.g. scientific literature, patents and intellectual property, and biology). Representative examples include PatentBERT [15], DistilBERT [16], BioBERT [17], DocBERT [18], K-BERT [19] and SciBERT [20]. More recently, these models have also been applied in multi-modal settings involving both text, and visual modalities, such as video [21,22].

Owing in part to the close connection between language and cognition [23,24], a growing body of research is seeking to deduce the cognitive abilities (or lack thereof) of LRMs [25,26]. There are both theoretical and practical reasons for this interest. The latter is important because these models are continuing to be integrated into, or otherwise used for, real-world applications and architectures in multiple enterprises and domains [27–30]. The former is also important and may be attributed to the empirical success and rapid advancement of these models. Particularly, as these models continue to get larger, and are proving to be capable in ways that had not been initially conceived [31], there is rising interest in their fundamental properties, such as the dependence of their performance on size and number of parameters [32], their robustness [33], including susceptibility to various flavours of adversarial attacks [34], and the amount of knowledge ‘encoded’ into their learned representations [35]. This line of research is detailed further in §2, where we discuss related work.

Along these lines, it is unclear if LRMs can be trusted to make approximately rational decisions, even when the outcomes are defined but are uncertain. In both behavioural and decision science, a number of experiments over the years have sought to test this ability in humans [36–39]. Classic work by Kahneman and Tversky showed, for instance, that people tend to exhibit loss aversion (prospect theory), and are not completely rational [40,41]. Despite this sensitivity to loss, however, prospect theory still predicts that an ordinary person would still choose gain over loss, if the gain substantially outweighs the loss in an equi-probable bet. Furthermore, people would not willingly choose to lose, if there was no possibility of gain attached to the choice. It is an open question whether LRMs take the same ‘common-sense’ approach to making decisions, or (by contrast) are prone to making decisions that would be considered extremely irrational in humans, such as actively pursuing loss (or to a lesser extent, zero gain) when a clear alternative is available with minimal risk.

This article proposes a detailed set of research questions (RQs) for empirically investigating the rational decision-making abilities of established transformer neural network-based LRMs. Inspired by long-standing work on behavioural science research mentioned above, the problem of making rational decisions is framed as one of testing a given model’s ability to think in bets and choose the outcome with the maximum expected gain. Specific contributions are enumerated below:

(i) We propose a novel set of RQs for understanding LRMs’ rational decision-making abilities. We construct and present a robust set of decision-making and preference elicitation benchmarks for empirically investigating these RQs. The benchmarks are designed to actively pre-empt common issues with such models, such as superficial pattern matching (shortcut learning) and dataset bias, which has also been found to be severe in other deep learning applications, such as computer vision [42–44]. To the best of our knowledge, this is the first attempt to quantify LRMs’ ability to make rational decisions by experimentally probing their capacity to ‘think in bets’.
(ii) We present a detailed and replicable methodology underlying the experimental study for investigating the presented RQs. Our methodology aims to control for several important factors that could serve as explanations for subsequently observed findings. Where applicable, we present new evaluation protocols to address such issues.
(iii) We conduct an extensive experimental study investigating each of the proposed RQs. Each study is accompanied by a detailed set of statistics, and appropriate baselines. We conduct the study on four transformer-based LRMs that are established models already being incorporated in several industrial products and services. As noted earlier, we also emphasize robustness (through multiple evaluation metrics and statistics) in the experimental design itself. Using multiple evaluation metrics, we find that our core conclusions are largely consistent with one another.

In this paper, our primary focus is on understanding and evaluating LRMs’ rational decision-making ability through a rigorously designed methodology and detailed experiments. We do not consider the related, and important, problem of modifying these models, or presenting new approaches, to improve the LRMs’ ability to make more rational decisions. That line of research, which builds upon the results presented herein, is left for future research.

The rest of this article is structured as follows. Section 2 discusses relevant related work, while §3 enumerates the specific RQs that fall within the scope of this article. Section 4 details the materials and methods underlying the study, and §5 follows with the key results and findings for each of the RQs listed earlier. Section 6 contextualizes these results with a broader discussion. Section 7 provides some guidance on promising avenues for future research before concluding the article.

## 2. Related work

This article is primarily influenced by two broad bodies of research: fundamental research on transformer-based LRMs, and experimental studies investigating the properties of these LRMs. The latter area is especially relevant to our goals. We discuss each of these in turn below, with specific focus on work that is best related to our research objectives. Additionally, our experimental methodology, and the manner of benchmark construction, are influenced significantly by decision-making experiments in the behavioural sciences [36,39,41]. However, these experiments were mainly conducted on humans (and at times, in domain-specific settings such as science [37]). This article seeks to follow similar principles in benchmark construction, but applies the benchmarks on LRMs. Because the article considers LRMs as the primary objects of study, we begin by describing some key models that are also employed in the empirical study in this article, followed by detailing other recent work on understanding the properties of these models, and transformer-based models more generally.

### 2.1. Transformer-based language representation models

As we noted in the previous section, LRMs, which are also called neural language models, have achieved great success on a variety of natural language understanding tasks over the last half decade. An early and influential LRM is BERT, which uses a novel attention mechanism to obtain rich ‘pre-trained’ representations of language from a large corpus of text. Pre-training is followed by task-specific ‘fine-tuning’ that allows it to get state-of-the-art performance (at the time) on specific tasks, such as question answering, without requiring full re-training from scratch. In experiments, BERT was found to obtain a score of 80.5% on the general language understanding evaluation (GLUE) benchmark [45], an improvement of 7.7% over the previous best performing model. Similarly, it achieved an F1 score of 93.1% on the Stanford question answering dataset (SQuAD) v. 1.1 benchmark [46] and 83.1% on SQuAD v. 2.0 [47], with improvements of 1.5% and 5.1% compared with the previous leading model, respectively. Moreover, the pre-trained BERT model was released publicly and is amenable to fine-tuning on other tasks. We draw on this capability in the proposed work.

A more mature version of BERT is the robustly optimized BERT pretraining approach (RoBERTa) [48]. RoBERTa is structurally the same as BERT. However, RoBERTa improves the training process on some key fronts, such as a bigger batch size, more extended sequence and longer training. RoBERTa also removes the next sentence prediction objective and introduces the dynamic masking strategy. Therefore, compared with BERT on their published GLUE and SQuAD performance, RoBERTa shows significant improvements and obtains new state-of-the-art results on four of the GLUE tasks. Like BERT, the pre-trained RoBERTa model was released and is amenable to being fine-tuned.

A more advanced version is the decoding-enhanced BERT with disentangled attention (DeBERTa) [9]. DeBERTa is structurally similar to BERT and RoBERTa. DeBERTa also introduces several novel techniques to improve performance even further. First, DeBERTa uses a disentangled attention mechanism, where two vectors are used to represent the content and relative position of each word, and correspondingly, disentangled metrics are used to compute the attention weights. Second, during pre-training, DeBERTa uses an enhanced mask encoder to combine the absolute positions in the decoding layer to predict the masked tokens. These novel methods improve DeBERTa’s performance on benchmark tasks. Compared with the RoBERTa-large model, DeBERTa improves on SQuAD v. 2.0 by 2.3%, on multi-genre natural language inference (MNLI) [49] by 0.9%, and on large-scale reading comprehension dataset from examinations (RACE) [50] by 3.6%. The pre-trained DeBERTa is also publicly available and amenable to being fine-tuned.

Another version is the transformers for longer sequences (BigBird) model [51]. BigBird deploys a sparse attention mechanism to reduce the quadratic dependency on sequence length, to linear dependency. The quadratic dependency on sequence length (in terms of memory) is one of the core limitations of transformer-based models, and necessary because of the full attention mechanism. As a result of its novel sparse attention mechanism, BigBird can handle up to 8× longer sequences using similar hardware, while demonstrating impressive improvements on question-answering benchmarks.

Although the four models above are cited as representative examples, LRMs have continued to advance and become ever larger even in the last 2 years. Examples of recent LRMs include the generative pre-trained transformer 3 (GPT-3) [3], the language models for dialog applications (LaMDA) [52] and the scaling language modelling with pathways (PaLM) [53]. However, due to their track record over the last 5 years, and their more manageable size, BERT-based models have tended to be incorporated in real-world systems, including within Google’s own search engine [54]. Therefore, for the experimental studies in this article, we use the four models detailed earlier (BERT, RoBERTa, DeBERTa and BigBird). Our benchmarks and methodology are applicable to other models that are capable of natural-language question answering, but we leave an investigation of the bigger models for future research.

Prior to the emergence of the transformer-based LRMs, the state-of-the-art text embedding method was the skip-gram-based FastText model [55,56]. By using the morphology of words, FastText acquires word representations as bag-of-character n-grams by pre-training on large corpora. Experimentally, it has been shown to achieve competitive results compared with other traditional word-level neural models (including those based on convolutional neural networks and recurrent neural networks) [57,58]. Apart from the four LRMs described above, we also adapt FastText as an additional (non-transformer) baseline model to investigate our RQs.

### 2.2. Understanding the properties of language representation models through experimental studies

Owing to the success of such LRMs, a recent line of work has emerged on understanding their properties using rigorous empirical methodologies, many inspired by research first conducted in the behavioural sciences. Prior work following BERT, for instance, has proposed approaches to better study the knowledge encoded within these deep transformer-based LRMs. Examples include ‘fill-in-the-gap’ probes for understanding the masked language model facility in LRMs [59,60], probing of other classifiers that take different BERT representations as their feature-inputs [61,62], deeper analysis of the self-attention weights in the LRMs [63,64], and even a checklist-style approach for comprehensively evaluating the linguistic abilities of a BERT-based model [65]. Detailed evidence suggests that BERT-based models seem to be encoding a ‘hierarchy’ of linguistic features, with surface features at the bottom, syntactic features in the middle and semantic features at the top [66]. Using massive corpora of pre-training text, the model is able to learn such a hierarchy implicitly without requiring explicit training labels.

While some work has found that information can be recovered from BERT’s token representation [67], the model still has trouble ‘understanding’ concepts that are relatively natural to humans, such as negation and basic numeracy [25]. Like many other machine learning models, the model can also be overly confident in some of its inputs, and is susceptible to problems of both generalization and adversarial attacks [34,43,68–70]. Furthermore, several experiments have demonstrated that, although BERT effectively encodes information about relations, entity types, relations, semantic roles, as well as proto-roles, it can lose some of its robustness in the face of basic named entity replacements [26].

This article contributes to this line of work by specifically investigating preference elicitation and rational decision-making abilities of such LRMs. We address the latter by posing bet questions to an LRM, and assessing its ability to think probabilistically, in terms of expected gains or losses, both when it is exposed to example bets (and allowed to ‘fine-tune’ on them) and when it is not. To the best of our knowledge, this is the first study to propose such an investigation, although there has been recent work on applying LRMs to the sequential decision-making problem [71], which is reminiscent of planning (rather than behavioural decision-making, as studied herein). However, much more recently, language models have been playing a growing and prominent role in cognitive science research [72,73], and the methodology and results presented herein are intended to complement this research. In general, there is more interdisciplinary focus today on developing a better understanding of complex AI models as these models are getting more applied in industry and society. In turn, such interdisciplinary investigations can enable researchers to build practical systems that fulfil more diverse needs. An example of such work is [74], which aims to do sentimental analysis in low-resource settings. Other work explicitly seeks to minimize inaccuracies that might arise due to differences in (for example) gender [75] or ethnicity [76]. A complete review of the growing ‘AI for social good’ movement is beyond the scope of this article; we recommend surveys such as [77] for the interested reader.

## 3. Research questions

This article proposes to investigate three specific RQs, the first of which investigates the preferences of neural language models when provided with pairs of items that are of ‘high’ or ‘low’ value, while the other two investigate their ability to make rational decisions under different assumptions and experimental conditions.

(i) *RQ1 (preference elicitation)*. Can LRMs be trained to prefer a high-value item (e.g. a diamond) over a low-value item (e.g. a plastic pen), where value is understood in common-sense economic terms?
(ii) *RQ2 (thinking in bets without task-specific fine-tuning)*. Are LRMs able to rationally bet on outcomes with higher expected gain without first being fine-tuned on such bet questions?
(iii) *RQ3 (thinking in bets with task-specific fine-tuning)*. Are LRMs able to rationally bet on outcomes with higher expected gain after being fine-tuned on such bet questions?

While the first question is not (in itself) central to the goals of this work, it is an important prerequisite for investigating the other two questions. This is because the concept of rationality is linked to an agent’s belief, or expectation, about value. For example, if an agent believes that an object A is more valuable than B, then given a bet with equi-probable outcomes (such as a coin toss), it is rational for the agent to ‘bet’ on the outcome with A as the prize. In other words, the agent’s preference of A over B influences our judgement of whether it subsequently makes a rational bet or not. Hence, understanding whether (and under what conditions) the language model’s preferences align with our own is an important RQ to address prior to investigating the model’s ability to bet on rational outcomes. In exploring this question, we also compare the trained model’s ability with that of a ‘default’ LRM that has not been trained specifically for preference elicitation (but that still performs well on general common-sense question-answering tasks) to quantify the effect of training itself on preference elicitation.

The second question directly considers the issue of whether LRMs are able to think in bets without first being fine-tuned on bet questions. As discussed subsequently, we refer to this as ‘task-specific fine-tuning’ wherein a model has been fine-tuned on a training set of questions that mirrors the purpose of the RQ, compared with the ‘default’ version, as mentioned above. However, we also investigate whether an LRM that has been fine-tuned for the preference elicitation task (RQ1) and performs well on it, is able to ‘naturally’ think in bets, even without first being fine-tuned on bet questions.

The third question investigates whether the model is able to think more effectively in bets, both in absolute terms and relative to RQ2, once it undergoes such task-specific fine-tuning. Although not stated directly in the question itself, we also investigate the LRMs’ generalization on bet questions that are structurally similar but have different surface form compared with questions that were used for task-specific fine-tuning. By structurally similar, we mean that the mathematical form of the bet, including the number of outcomes, and the probabilities associated with the outcomes, remains the same. An example would be two bets of the form where one involves tossing a coin and the other involves randomly picking a card (from the standard 52-card deck). An outcome is then associated with whether the coin comes up heads or tails (for the former), or whether the randomly picked card is black or red (for the latter). Assuming that the bet-wager and outcomes are identical in the two cases, the bets described above are structurally similar, but with different natural language descriptions associated with them. The third question attempts to quantify whether, and to what extent, the LRMs’ decision-making ability erodes when the surface form of the question changes to one that it has not seen during fine-tuning.

As noted in §2, our RQs are heavily inspired by similar experiments in decision science and psychology, many of a classic nature [40]; namely, we seek to understand a language model’s rationality, and its ability to seek outcomes with maximum expected gain, by studying its preferences on a carefully designed set of prompts. It is important to design the prompts to control for a range of problems that are known to occur with such language models, including their sensitivity to format [78], and their propensity to achieve high performance through advanced, but ultimately superficial, statistical pattern matching [42]. Hence, the experimental design, benchmark construction and evaluation methodology are critical elements of the study, and are extensively detailed in the following section.

## 4. Material and methods

### 4.1. Language representation models

As discussed in §2, many of the recent advances in LRMs are based on transformer neural networks [79]. In some instances in the literature, these are referred to as language representation *learning* models, or even *neural* language models. We adopt the uniform terminology of LRMs in this article, with the understanding that we are primarily interested in the recent neural models.

LRMs, such as BERT [1] and the GPT [2] series of models, have been found to generalize on an impressive range of language understanding tasks, including machine translation and question answering [80–83]. In the remainder of this article, we uniformly use the term LRM to refer to the models that are used to answer ‘multiple-choice’ prompts or questions by selecting one answer from a set of candidate choices.

To be applied to specific natural language processing (NLP) problems, these models, which are *pre-trained* on a large corpus of text before being publicly released, are typically also *fine-tuned* on an additional smaller dataset to optimize them for the task at hand. For example, if BERT were to be applied to the problem of named entity recognition (NER; automatically extracting named entities, such as people, places and organizations, from text), the pre-trained version would have to be fine-tuned on a ‘training’ set of clearly defined NER inputs and outputs. Fine-tuning takes a much smaller amount of time compared with pre-training. This makes pre-trained LRMs a powerful asset in the NLP literature because these models can be used as a ‘base’ model for a wide range of tasks and datasets, a facility that we rely upon for the decision-making experiments herein.

Owing to its training on a large body of text, the pre-trained model can be fine-tuned to ‘score’ a natural language sentence based on its likelihood of being a plausibly constructed sentence. The higher the score, the more plausible the sentence. Impressively, the score correlates not just with real-world syntactic usage but also plausible semantics, depending on the background corpus on which the model was pre-trained. For instance, if the model was pre-trained on a general corpus, such as Wikipedia or Google Books, nonsensical sentences would tend to be given much lower scores by the model. However, in some cases, the pre-training corpus is domain-specific, such as with the BioBERT pre-trained model [17] or social media-based pre-training [84]. Such models will normatively assign higher scores to biological and social media sentences, respectively. We only use models in this article that were pre-trained on general corpora, such as Wikipedia and news articles.

Although there are many viable transformer models (and their variants) available at the time of writing, we selected four models for our studies, first introduced and discussed in §2: BERT [1], RoBERTa [48], DeBERTa [9] and BigBird [51]. We emphasize again that RoBERTa is fundamentally similar to BERT, but is often treated separately because of its (much) higher performance over the original BERT release owing to its robust optimization, and other important engineering innovations [48]. Our rationale for selecting these four LRMs is that these are established models that have been rolled out in a range of commercial and outward-facing products, including the Google search engine [54] and Amazon Web Services [85]. Many technical and domain-specific variants of these models have also been developed and deployed, including SciBERT [20], BioBERT [17] and AlBERTa [86].

Each of these four models has a publicly available pre-trained version, but can also be fine-tuned on additional question-answering datasets. Since the training dataset used for fine-tuning depends on the experiment and research hypothesis, we specify the dataset used for fine-tuning when discussing the experimental methodology for the corresponding research hypotheses. Next, we describe the specific manner in which each of these LRMs can be applied to the multiple-choice question answering (MCQA) problem, which is of central interest in this article.

### 4.2. Multiple-choice question answering using LRMs

In this section, we introduce some basic formalism on MCQA instances, and on the specific methodology that we use to obtain an LRM’s prediction for a given instance. An MCQA instance formally consists of two elements: a ‘question’ prompt *q* and a set *C* of *n* ‘answer’ choices {*c*_1_, *c*_2_, …, *c*_*n*_}. We assume, without loss of generality, that each of the choices and *q* is represented as a string. Furthermore, it is usually assumed that exactly one of the choices in *C* is ‘correct’. Given a set of MCQA instances (referred to as an MCQA *benchmark*), the goal of a question-answering system, such as an appropriately fine-tuned LRM, is to predict the correct choice for the prompt.

One approach by which an LRM can be made to answer an MCQA instance is as follows. First, the question prompt *q* is concatenated with *each* of the choices *c*_*i*_ in turn. This yields *n* question–answer pairs, where a pair *p*_*i*_ = *concatenate*(*q*, *c*_*i*_). Next, each of the *n* pairs is fed into the model in turn (i.e. independent of one another) during the fine-tuning phase, when the correct answer can be revealed to the model. Specifically, if the *c*_*i*_ used to form *p*_*i*_ is the correct choice, *p*_*i*_ is labelled as 1 (otherwise it is labelled as 0). Given such a ‘training’ set, the model is fine-tuned to minimize the cross-entropy loss as is standard for MCQA problems.

The fine-tuned model can then be evaluated using a similar methodology on unseen MCQA instances, for which it needs to predict the correct answers.

First, we convert such a ‘test’ MCQA instance to a similar input structure, as used during fine-tuning, by concatenating each choice *c*_*i*_ to the question prompt *q* (to obtain pair *p*_*i*_). Next, the model is provided with each *p*_*i*_ *independently*, and outputs a score for each such pair. The score is assumed to be proportional to the model’s belief in that pair being labelled as 1 in the underlying ground-truth. Because the model’s score is not necessarily normalized, we use the sigmoid function,^1^ to normalize each score to the range [0,1]. Although just the highest-scoring choice could be selected as the model’s prediction, there is an alternative mechanism available for selecting (and evaluating) the predictions in a decision-making context, as later discussed. For this reason, it is more appropriate to say that, depending on the specific experiment and RQ, a *predicting function* is applied to the *n* normalized scores (corresponding to the *n* choices in an MCQA instance) to yield the model’s prediction for that instance.

As a concrete example, consider the MCQA instance in table 1. The fine-tuned LRM would be given each question–answer pair in turn. If the predicting function is simply to select the choice for which the model outputs the highest score, and this choice happens to be ‘this statement is true: aeroplane is more expensive than pen’, then the model would (correctly) select the choice ‘aeroplane is more expensive than pen’ as its prediction, given the prompt ‘this statement is true:’.

**Table 1.** An example of a multiple-choice question answering (MCQA) instance (prompt and choice-set) and the concatenated pairing of the choices in turn that is used to evaluate the LRMs in this paper.

prompt	choice	question–answer pair
this statement is true:	aeroplane is more expensive than pen	this statement is true: aeroplane is more expensive than pen
	pen is more expensive than aeroplane	this statement is true: pen is more expensive than aeroplane
	aeroplane and pen have the same value	this statement is true: aeroplane and pen have the same value

We place no constraints at present on the predicting function: it may select zero, one, or more than one, choice, as the prediction for an MCQA instance. In the next section, we describe two plausible choices for the function, one of which is to just select the highest-scoring choice.

An alternative approach to fine-tuning a model for question answering is to concatenate the question prompt *q* with all of the choices (*c*_1_, *c*_2_, …, *c*_*n*_) together, along with a separator between each of the choices, i.e. (1, 2, …, *n*). This yields a single ‘complete’ multiple-choice question, which is denoted as *mcq* = *concatenate*(*q*, 1, *c*_1_, 2, *c*_2_, …, *n*, *c*_*n*_). Instead of 1 or 0, the expected output (or label) of the model should be the string of the correct choice, denoted as *c*_*T*_. The actual fine-tuning process is similar in that the model generates string output given the *mcq*, and the cross-entropy loss between *c*_*T*_ and this string output is used in the optimization. However, while this can work well for ‘generative’ question-answering problems, models such as BERT are better suited for discriminative problems (of which the MCQA problem is one) and typically adopt the first approach. While language models, such as UnifiedQA, have also been applied to generative QA problems [12], we leave a generative evaluation of a model’s decision-making ability for future work and assume the first or ‘discriminative’ approach in the rest of the paper.

#### 4.2.1. Predicting functions: standard method and threshold method

An obvious choice for the predicting function that we had mentioned briefly earlier, applicable when exactly one ‘correct’ prediction is desired from the model, is to select the choice *c*_*i*_ corresponding to the highest normalized score. We call this function the *standard method*, as it is the method favoured in much of the QA literature where exactly one choice is correct and all other choices are incorrect. An example presenting the method in action was earlier presented in the context of table 1.

However, in the decision-making benchmarks that are considered in this article, the assumptions about correctness are more nuanced. For example, while our benchmark construction (subsequently described) always guarantees that there is a single ‘optimal’ answer, it is not always the case that all other answers are equally suboptimal. Some choices provided with a bet question may be associated with positive expected gain (even though they may not be associated with the *highest* positive expected gain, which would be the case for the optimal answer) while others may be associated with zero, or even negative, expected gain. An important empirical objective in this paper is to determine whether the model is able to understand these differences, especially when it selects suboptimal choices.

Hence, to more holistically evaluate the model’s decision-making ability, we also consider a second predicting function called the *threshold method*. As the name suggests, instead of simply selecting the choice with the highest score, this method involves selecting all choices (as predictions) that lie *above* a threshold. Furthermore, if no choice lies above the threshold, the model refuses to yield a prediction. In fact, given *n* choices in an MCQA instance, it is easy to show that a model can theoretically yield the power-set (of size 2^*n*^) of selections of choices, of which the empty set is one extreme possibility, and the complete set (selecting all choices) is the other extreme possibility.

In principle, this methodology is similar to that employed in a wide range of practical machine learning applications that require the careful selection of a threshold in order to optimize a non-trivial quality metric (such as F1-score) on a multi-label problem [87]. Similarly, when describing the benchmark construction, we systematically consider how to evaluate the quality of a model when using this method, but for now, it suffices to say that there is more than one reasonable way to construct a ‘ground-truth’ against which to evaluate an LRM’s (multi-label) predictions when using the threshold method. We define such a ‘binary’ ground-truth as stating which of the 2^*n*^ possible power-set predictions should be considered correct or incorrect, on the basis of which an accuracy metric can always be computed for a model. For example, one choice of ground-truth might consider as correct any combination of choices (that a model selects, using the threshold method) that yields positive expected gain, while another (less-conservative) ground-truth may only decide to test for consistency (i.e. it may only penalize a set of selected choices that are directly contradictory, such as ‘bet on heads’ and ‘do not bet’). We also provide the rationale and specific rules governing ground-truth construction for a corresponding benchmark.

Because there is more than one way to judge the quality of such multi-label predictions, an ‘optimal’ choice for this threshold is not a fixed value, and depends not only on the manner (including the choice of ground-truth) in which the model’s performance is being judged, but also on the benchmark itself. Furthermore, a threshold that works well for one model (and for a given quality metric) may be suboptimal under a different experimental condition. For all of these reasons, instances in each of our benchmarks are always partitioned into a train, development and test set. Where applicable, the train set is used for fine-tuning, and without exception, the test set is always used for evaluating all models under a given experimental condition to ensure a fair comparison. Similarly, when using the threshold method as a predicting function, the development set is always used to determine the ‘optimal’ threshold, given an LRM, and choice of ground-truth against which the LRM’s predictions will be judged.

To discover such a threshold, which is technically a *hyperparameter* that takes values in [0,1], we first do a simple grid search in that range using increments of 0.01. In the event that more than one threshold value achieves the maximum performance on the development set, we select the median of the values that achieve this maximum as the expected optimal threshold for that experiment.

#### 4.2.2. Fine-tuning language representation models for multiple-choice question answering

As described earlier, the pre-trained versions of the LRMs need to be fine-tuned on appropriate ‘training’ sets before applied on tasks with a specific structure. MCQA is an example of such a task (and is the primary focus of this article) but other common examples in the NLP literature include named entity recognition [88] and information extraction [4]. The four pre-trained LRMs that we fine-tuned for the MCQA experiments in this paper are BERT, RoBERTa, DeBERTa and BigBird, all of which were introduced earlier. Note that these LRMs have variants in the HuggingFace repository that we used for accessing and fine-tuning the models. The specific variants that we used are BERT_BASE_ [89], RoBERTa_BASE_ [90], DeBERTa_BASE_ [91] and BigBird_BASE_ [92].

Although we could directly fine-tune each of these pre-trained models on the train set of the MCQA benchmark that we construct, one of our empirical goals is to understand whether such models, if fine-tuned on a ‘general purpose’ MCQA benchmark, are able to exhibit reasonable decision-making ability as a natural consequence of such fine-tuning. One such benchmark that is widely used in the community is the situations with adversarial generations (SWAG) dataset [81]. SWAG is a common-sense benchmark that contains MCQA instances on grounded common-sense inference and physically grounded reasoning.

By fine-tuning each of the four LRMs on SWAG, we can test whether good performance on such common-sense tasks necessarily entails good performance on decision-making and preference elicitation problems expressed using everyday language and objects. Since these SWAG-based fine-tuned models form a natural basis of comparison with models that are further fine-tuned to handle our decision-making benchmarks, we refer to them as the *default* models. For example, the *default BERT* model is used to refer to the BERT model that has been fine-tuned on the training partition of the SWAG benchmark.

For the fine-tuning itself, we use a batch size of eight and fine-tune each of the four models for three epochs (and a total of 27 500 steps) each on the 73 546 MCQA instances in the SWAG training set, using a learning rate of 5 × 10^−5^. Following the fine-tuning, we verified that, on the SWAG validation set, the accuracy of the default BERT, RoBERTa, DeBERTa and BigBird model is 77%, 79%, 85% and 81%, respectively. These results are consistent with previously published results and confirm that the models are indeed able to achieve good performance after fine-tuning. Finally, we uniformly use a batch size of 32 and a learning rate of 5 × 10^−5^ for any other (i.e. non-SWAG) fine-tuning, described below, in our experiments.

Because the default model can be *further* fine-tuned on another MCQA benchmark with a similar structure, we use it as the ‘initialization’ for fine-tuning an LRM on the train set of our own benchmark (which depends on the specific RQ). We mnemonically refer to such a model as a *task-specific fine-tuned LRM*. Since each of our benchmarks is always partitioned into train, development and test sets, we use the train set for the actual fine-tuning, and we stop the fine-tuning once the model has achieved an accuracy of 90% on the development set. A key point that we emphasize here is that, unlike the default model (of which there is only one unique model per LRM, since it is never fine-tuned on a decision-making benchmark), the task-specific fine-tuned model depends on the benchmark that was used for further fine-tuning. Hence, there is a unique task-specific fine-tuned model per LRM *and* benchmark. Finally, when evaluating a (default or task-specific fine-tuned) model using the threshold method, the development set is ‘re-used’ for determining the expected optimal threshold for that model using the grid-search procedure described earlier.

### 4.3. Benchmark construction

This section describes the construction of benchmark QA datasets used for investigating the RQs. These datasets have also been uploaded for public access to facilitate replication and further investigation [93].

#### 4.3.1. High-value and low-value sets of items

We manually create two sets of ‘high-value’ and ‘low-value’ items to facilitate our experiments. These items are tabulated in table 2. We created these sets with the intent that any regular person would be able to distinguish these items fairly easily, especially if asked to do so in terms of (difference in value in) dollar amounts. We recognize that ‘value’ can be understood in different ways, and can even be contextually dependent, e.g. a ‘low-value’ item can always become high-value in the right set of circumstances, and vice versa. We adopt a common-sense, everyday view here, with value best thought of in economic terms. This is a standard premise in the decision-making literature, going back to the pioneering behavioural psychology experiments devised by Tversky & Kahneman [94]. Also, it is the *distinction* (or relative difference) between high and low value items that we control for in our experiments, not the absolute value of these items. Furthermore, in our experimental design, we take into account the potential concern that an LRM may not understand ‘value’ in quite the way described above. We present questions using several different *templates*, including making the economic aspect of value explicit in one of these templates. This construction is discussed further in the next section.

**Table 2.** High-value items and low-value items, divided into train, development and test sets.

*train set*	
high-value items	airport, airship, bike, bicycle, bus, camera, gold, supercar, refrigerator, jewellery, hotel, horse, guitar, tank, helicopter
low-value items	baseball, bread, brush, chair, chocolate, vegetable, soup, shirt, orange, knife, fish, cookie, cigarette, honey, newspaper
*development set*	
high-value items	watch, ipad, phone, TV, telescope
low-value items	egg, apple, soda, toothbrush, toothpaste
*test set*	
high-value items	car, house, diamond, aeroplane, computer
low-value items	pen, paper, water, slipper, sock

The high-value items are partitioned into ‘train’, ‘development’ and ‘test’ high-value sets (and similarly for the low-value items). The roles of the train and development sets were described earlier when the fine-tuning procedure for LRMs was discussed. Unless explicitly noted, the outcome of the experiment will be reported using the test set. Importantly, this is true even when we are not fine-tuning the model on our train set, i.e. when we are using a default model.

This allows us not only to use *paired* statistical significance testing when applicable, but to use common item-sets as the basis for all experiments. This ensures that any differences observed between experiments cannot be explained through differences in benchmarks used during testing.

One concern that might arise when we use such high- and low-value items to experiment on LRMs is that the items’ value is often contextual and the LRMs might have a preference different from the one we had intended (i.e. that a high-value item should be preferred over a low-value item). The first RQ (preference elicitation) is designed to test whether this is indeed the case and if the LRMs prefer high-value items to low-value items, on average.

#### 4.3.2. Value questions

Given the high-value items and low-value items described in the previous section, RQ1 seeks to determine whether an LRM prefers (at least, on average) a high-value item over a low-value item. One manner in which we can do this is by prompting an LRM to choose the more ‘valuable’ item from a given pair of items (one of which could be high-value and the other of which could be low-value). More precisely, we could design a *template*, defined as a partial instance with placeholders that is converted into an actual instance by being appropriately *instantiated*. An example of a template would be the partial instance: ‘This statement is true: (a) [h] is more expensive than [l] (b) [l] is more expensive than [h] (c) [h] and [l] have the same value’ Here, [h] and [l] are placeholders for a high-value and low-value item from table 2, respectively. To differentiate between a template and its instantiated equivalent, we refer to an instantiated template as a *value question*.

To reasonably control for format and potential different interpretations of ‘value’, we designed four different templates for determining LRMs’ preferences. Controlling for format is motivated by the fact that LRMs are known to be sensitive to the mode of presentation of a prompt and answer choices, as evidenced in previous work [95]. Using only one template runs the risk of drawing conclusions about a model, even though the model itself may be quite capable of distinguishing between differently valued items if the inputs were presented in a slightly different format. At the same time, it is obviously not feasible to try *every* possible format for investigating whether a model truly prefers a higher-value item ‘h’ to a lower-value item ‘l’. Hence, we decided on a pragmatic compromise by constructing four templates that are structurally dissimilar; yet, consistently likely to yield results that would allow us to confidently distinguish between LRMs’ preferences.

These four templates are listed in table 3, along with an example instantiation using a high-value and low-value item combination from table 2. While the most important difference between the templates is the manner in which answer choices are presented (*Boolean* versus *choice*), another important difference is that two templates use the word ‘valuable’, while two others use the word ‘expensive’ (*valuable* versus *expensive*). We anticipate that using the four templates derived from these two formatting controls will allow us to gain a more robust understanding, and conduct consistency checks, of the differences between LRMs’ preferences for pairs of (high-value and low-value) items sampled from table 2. For each template, there are 225 MCQA instances if instantiated using the train set items and 25 MCQA instances if instantiated using the development set or test set items.

**Table 3.** The four templates, mnemonically named, along with an example instantiation (value question) that respectively substitutes an actual high-value item and low-value item for [h] and [l] in the corresponding template.

name	template	example instantiation (value question)
Boolean expensive	this statement is true: (a) [h] is more expensive than [l] (b) [l] is more expensive than [h] (c) [h] and [l] have the same value	this statement is true: (a) car is more expensive than pen (b) pen is more expensive than car (c) car and pen have the same value
Boolean valuable	this statement is true: (a) [h] is more valuable than [l] (b) [l] is more valuable than [h] (c) [h] and [l] have the same value	this statement is true: (a) car is more valuable than pen (b) pen is more valuable than car (c) car and pen have the same value
choice expensive	from [h] and [l], choose an item that is more expensive: (a) [h] (b) [l] (c) the same	from car and pen, choose an item that is more expensive: (a) car (b) pen (c) the same
choice valuable	from [h] and [l], choose an item that is more valuable: (a) [h] (b) [l] (c) the same	from car and pen, choose an item that is more valuable: (a) car (b) pen (c) the same

Note that a value question associated with a high-value item ‘h’ and a low-value item ‘l’ can have different ground-truths depending on the choice of predicting function assumed:

(i) *Standard method.* For each value question, the only correct choice in the ground-truth is the one implying that (depending on the template) ‘h’ is more *valuable* or *expensive* than ‘l’. All other choices are incorrect.
(ii) *Threshold method.* Recall that, per this method, the model may select zero, one or multiple (including all) choices as its prediction. Such a multi-label prediction can always be represented as a *set* of choices. An appropriate, binary ground-truth must operate at the level of sets, rather than choices. Since each value question always has three choices, there are 2^3^ = 8 possible predictions. We design three different ground-truths to determine which of these eight predictions should be considered as ‘correct’ or ‘incorrect’, each of which successively relies on a ‘broader’ definition of correctness.
  (a) *Normal.* Analogous to the ground-truth used for the standard method, a model’s prediction is deemed as correct if it returns the set that contains only the one correct answer per the standard method (i.e. depending on the template, that ‘h’ is more valuable or expensive than ‘l’). Note that accuracy, measured using this ground-truth, can theoretically be different from the accuracy measured using the standard method. One reason (although not the only one) is that the maximum-scoring answer may have a score that is below the (empirically determined) threshold, in which case no answer is selected and the empty set is returned.
  (b) *Weak normal.* This ground-truth takes a broader view of correctness than the normal ground-truth. In addition to what the normal ground-truth deems correct, this ground-truth also rates the model’s prediction as correct if it selects both the correct answer (per the normal ground-truth) *and* the answer that states that the two items are equal in value. We interpret this output as the model predicting that the high-value item (in the question) greater than or equal to the low-value item. Any other combination of selected answers (as well as the empty set) beyond the two possibilities stated above would be treated as incorrect.
  (c) *Weak.* This ground-truth takes the broadest view of model correctness. Unlike the other two ground-truths, it does not test whether the model is outputting the correct value preference; rather, it tests whether the model is either contradicting itself or otherwise refusing to state a preference. Hence, only the following three predictions are considered as incorrect (and each of the other five possible sets are considered as correct): (i) the set containing all three choices, (ii) the empty set and (iii) the set containing the two ‘strict inequality’ choices (expressing simultaneously that the value of ‘h’ is strictly higher *and* lower than the value of ‘l’). Intuitively, (i) and (iii) imply a ‘contradiction’ while (ii) implies a non-decision that we treat as incorrect.
  Note that, because the three ground-truths successively rely on weaker notions of what counts as correct, the expected performance of a system that randomly selects from the eight possible sets correspondingly increases. Specifically, across each of the test sets used for evaluating RQ1 (regardless of template), expected random performance using the normal, weak normal and weak ground-truth is 12.5%, 25% and 62.5%, respectively. Expected random performance using the standard method is 33%, which is (expectedly) different from the normal ground-truth random performance, for the reason discussed earlier.

#### 4.3.3. Bet questions

While RQ1 investigates LRMs’ preference for high-value items over low-value items, RQ2 aims to go a step further by evaluating whether LRMs are able to use this knowledge, whether directly or indirectly, to think in bets. Similarly, RQ3 aims to evaluate whether the models, if fine-tuned on bet questions, are able to successfully think in bets that have not been seen before.

Both RQ2 and RQ3 require presenting to the model a bet question, with different *uncertain* outcomes (each of which is associated with an expected gain), to investigate the degree to which the model is able to make a rational bet. Although both RQs evaluate the model on bet questions presented during testing, the key difference between them, discussed subsequently, is that RQ2 does not use bet questions for fine-tuning, while RQ3 does.

We design the bet questions using a similar template-based methodology as the value questions. These templates contain placeholders for high- and low-value items (that can be instantiated using the items in table 2), but to expand and diversify the manner in which bets are presented to the model, we constructed questions using different bet *modalities*, mnemonically designated as *coin*, *card* and *dice*, and demonstrated in table 4. Importantly, all three modalities involve equi-probable outcomes and are expressed using natural (rather than mathematical) language. We refer to an instantiated bet template as a *bet question*. For each modality, there are 900 MCQA instances if instantiated using the train set items and 100 MCQA instances if instantiated using the development set or test set items.

**Table 4.** Templates expressing the three bet modalities used for evaluating both RQ2 and RQ3, that can be instantiated using a high- and low-value item (represented as [h] and [l], respectively). Note that a question cannot be instantiated using two high-value or two low-value items (i.e. if a high-value item is selected for the first placeholder, a low-value item must be selected for the second placeholder).

modality	question template
coin	if the coin comes up heads, then I win a [h]/[l]. If it comes up tails, then I lose a [l]/[h]. What should I do to maximize my expected gains? (a) I should bet on heads (b) I should bet on tails (c) I should not bet on either one
dice	if the dice comes up even, then I win a [h]/[l]. If it comes up odd, then I lose a [l]/[h]. What should I do to maximize my expected gains? (a) I should bet on even (b) I should bet on odd (c) I should not bet on either one
card	if I pick a card from a standard deck of cards, and the card is red then I win a [h]/[l]. If it is black, then I lose a [l]/[h]. What should I do to maximize my expected gains? (a) I should bet on red (b) I should bet on black (c) I should not bet on either one

These bet questions are based on some reasonable assumptions that are expected to hold in practice and are consistent across all questions. First, even though it is not directly mentioned in the question, any model that chooses to bet is assumed to wager some amount of money on the outcome it chooses. For example, in the case of coin questions, the model may choose to wager on either heads or tails (if it chooses to bet). Second, once the bet is executed and the outcome is known, the following standard reward structure is assumed: whatever outcome comes to pass, the win/loss event associated with the outcome (e.g. winning a watch if the coin comes up heads) will be executed, unless the model chose not to bet. Note, however, that in our experiments, we do not actually ‘simulate’ the execution of the bet, since we are only interested in expected values. Regardless of the executed event, however, if the model bet on the outcome that came to pass, it will receive its wagered amount back; otherwise it will lose its wagered amount.

Although we could have included the wagered amount explicitly, or made it a free parameter, doing so would have added the confounding element that the model, were it to perform badly, was doing so because of its limited ability to handle *explicit numeracy*. Studies have shown that numerical reasoning can be problematic for some language models, and that it may need special treatment [96,97]. To control for this, we rely on a construction that assumes (approximately) *balanced expectation*, i.e. the wager is an amount that lies roughly between the high- and low-value items, but is skewed slightly toward the lower end to allow for some outcomes to have positive expected gain associated with them. More technically, if we denote the value of a prototypical high- and low-value item as *H* and *L*, respectively, the wagered amount *X* is assumed to obey the inequality *L* < =*X* < =0.5 ∗ (*H* − *L*).

With the above assumption in place, we calculate expected gain for the different bet questions (reproduced in the appendix in tables 17 and 18), with ground-truths constructed accordingly. In all cases, this construction guarantees that there is always (exactly) one optimal-expectation outcome. However, depending on the question, there may be outcomes that have positive expected gain, but are not necessarily optimal. Furthermore, because we allow the model to choose not to bet, the optimal-expectation outcome is never negative; however, in some cases, not betting is optimal, with zero gain, since all other outcomes are associated with negative expected gain. As discussed in the next section, we evaluate the model under several different scenarios and metrics to gain a more comprehensive and robust understanding of its decision-making, rather than always expecting it to choose the one outcome that is strictly optimal.

Finally, we instantiate and design the templates in a way that minimizes the possibility of model-overfitting due to (potential) superficial pattern matching. One way in which we do so is by flipping the position of ‘win’ and ‘lose’ in the templates shown in table 4 to avoid a fixed outcome always being the optimal (or even positive expected gain) choice. Formally, given *m* and *n* high- and low-value test items, respectively, the total number of coin-modality test questions would be 2 × 2 × *m* × *n* = 4*mn*, and similarly for the other modalities. While the first doubling effect accounts for the win/lose swapping (not shown in the template), the second doubling effect accounts for the fact that the first [h/l] placeholder could have either a low-value or high-value item.

In some cases, not betting is clearly the rational choice. For example, considering the coin question template, if the model wins a low-value item and loses a high-value item, then under the assumption about the wager stated earlier, not betting is rational. In the next section, we formalize this intuition by presenting RQ-specific metrics that quantify LRMs’ ability to distinguish between these possibilities.

Similar to the value questions, the ground-truth for the bet questions depends on the choice of predicting function used during evaluation:

(i) *Standard method.* Because each choice is associated with an expected gain, the only correct choice is the one that maximizes expected gain. Each of the other two choices is deemed as incorrect. Note that the construction of the benchmark guarantees that there is always one unique choice with maximum expected gain (which is always non-negative).
(ii) *Threshold method.* Because the model now returns a set of choices (leading to 2^3^ = 8 possible predictions), each such prediction needs to be systematically assigned an expected gain. Note that, when the model returns the set consists of the two ‘I should bet’ choices (expressing that I should bet on both outcomes e.g. heads *and* tails) as prediction, we assume that the wagered amount is split equally between the selected choices, and we calculate the expected gain of such prediction accordingly. For all other prediction possibilities, we provide a detailed calculation of the expected gain in the appendix. While exactly one choice (per bet question) is always associated with an optimal gain, which has the highest expected gain among all the possible predictions, the other choices are not necessarily equally suboptimal. To investigate the LRMs’ decision-making abilities holistically, we design three ground-truths to determine the correctness of the eight multi-label predictions.
  (a) *Strict.* Similar to the ‘normal’ ground-truth in the benchmark used for RQ1, the ‘strict’ ground-truth only deems a model to have correctly answered a bet question if it returns the set that contains only the optimal choice (i.e. the choice with the highest expected gain). If the optimal choice is not in the set, or choices besides the optimal choice are in the set, the model’s prediction is deemed as incorrect. For reasons similar to those stated earlier, the accuracy measured using the ‘strict’ ground-truth can differ from the accuracy measured using the standard method.
  (b) *Positive gain.* This ground-truth measures the model’s ability to return a set, such that the choices in the set collectively yield a positive expected gain, when such a set exists. Calculations for which such predictions would have positive expected gain, under the wager assumption stated earlier, are provided in the appendix. Note that, for some bet questions, each of the eight possible predictions is associated with either zero or negative expected gain. Since there is no ‘correct’ prediction possible for such questions (when evaluating a model using this ground-truth), we exclude such bet questions when evaluating a model using this ground-truth. Furthermore, any set containing choices that are either collectively contradictory, as well as the empty set, is deemed as incorrect. Hence, the following predictions would always be considered incorrect: (i) the set containing all three choices, (ii) the empty set and (iii) the set containing the choice ‘I should not bet on either one’ *and* any one of the other two choices.
  (c) *Non-negative gain.* This ground-truth measures the model’s ability to return a set containing choices that collectively yield a non-negative expected gain (including zero expected gain). This ground-truth is the broadest of the three ground-truths discussed thus far. However, unlike the positive gain ground-truth, all questions have at least one correct set associated with them, since the choice of not betting is always associated with zero expected gain. Note also that the three scenarios listed for the positive gain ground-truth, whereby a prediction would always be considered incorrect if it implied non-decision or contradiction, also apply to this ground-truth.

Similar to the RQ1 benchmark, because the three ground-truths successively take a broader view of what predictions count as correct, the expected performance of a system that randomly selects from the eight sets, correspondingly increases. Specifically, the expected random performance using the strict, positive gain and non-negative gain ground-truths is 12.5%, 25% and 25%, respectively. The expected random performance using the standard method is still 33%.

Unlike cognitive research of similar nature, the ‘subject’ of our research is one or more LRMs and not a human being (i.e. the decision-making entity is a language model and not an actual person). Hence, this study does not qualify as human subjects research and is exempt from requiring formal ethical approval by an institutional review board. However, we are careful in this article to appropriately and cautiously qualify and interpret our findings, given that LRMs are continuing to be rolled into real applications and there is increased scrutiny about their capabilities.

### 4.4. Experimental set-up

#### 4.4.1. RQ1: preference elicitation

We use the value-questions benchmark described under *Benchmark construction* for investigating RQ1. The full benchmark comprises four different templates, each of which is instantiated using the train, development and test item-sets. We report results for the default and task-specific fine-tuned models, using both the standard method and the threshold method. We emphasize that, while there is a single default model per LRM, there is a task-specific fine-tuned model per LRM *and* per template. Hence, the full RQ1 evaluation involves a total of four default LRMs, and 16 task-specific fine-tuned LRMs.

We use the accuracy metric for reporting performance. A single accuracy estimate is reported for the standard method for each experimental setting (i.e. choice of LRM and template). By contrast, three accuracy estimates are reported for the threshold method, with each estimate corresponding to each of three ground-truths (normal, weak normal and weak). In a slight abuse of terminology, we use the name of the ground-truth itself to refer to the corresponding accuracy of the model being evaluated using that ground-truth.

We also report on the statistical significance of each result by using the one-sided *z*-test to evaluate whether the result is better than the expected random performance, i.e. the expected accuracy of a system that selects randomly from among the answer choices. For the standard method, the expected random performance is 33% for all experimental settings in RQ1, since only one out of three possible choices can be selected by any system being evaluated using the standard method (and exactly one choice is correct). For the threshold method, we similarly computed the fraction of correct answers for each of the three ground-truths, which would equal the expected random performance. We found the expected random performance for the normal, weak normal and weak ground-truth to be 12.5%, 25% and 62.5%, respectively. In the results, we use a maximum Type-I error rate *α* = 0.05 to confirm significance (in other words, the one-sided *p*-value, using the *z*-test, must not exceed 0.05 to be significant); however, the complete set of *p*-values for all tests are also reported.

In addition, we also adapt the skip-gram-based FastText model as a non-transformer baseline. We use a pre-trained FastText model [98] containing 2 million word vectors that were trained with sub-word information obtained from the Common Crawl. To use this model as a baseline, we first convert the multiple-choice question answering (MCQA) instance to the same format (described earlier) as used by the LRMs. Specifically, given an MCQA instance with *n* answer choices, we obtain *n* question–answer pairs by concatenating the prompt and each of the choices. Then, we use the pre-trained FastText model to tokenize each sub-word in a question–answer pair. We use the mean vector of each sub-word in a question–answer pair as the final vector representation of that pair. The question–answer pair that contains the correct choice is deemed as correct and labelled as 1, while the other question–answer pairs are deemed as incorrect and labelled as 0.

During an evaluation, a model must produce a probability estimate of a pair being labelled as 1. The pair with the highest such probability produced by the model on all question–answer pairs (derived from a MCQA instance) is then used as the model’s prediction for that MCQA instance. To obtain these probability estimates, for each template, we train a logistic regression model using the question–answer pairs from the MCQA instances instantiated using train set items. The ‘feature vectors’ input to the model are the FastText embeddings obtained using the averaging procedure described above. Next, we use the trained model to report a single accuracy estimate for that template (instantiated using test set items). In total, four accuracy estimates are reported, since there are four templates for RQ1.

#### 4.4.2. RQ2: thinking in bets without task-specific fine-tuning

We use the bet-questions benchmark described under *Benchmark construction* for investigating RQ2. The four default models used for RQ1 are also used for RQ2. Furthermore, since the (task-specific) fine-tuned models used for RQ1 behave similarly across the four templates (as the results for RQ1 will show), we only report results for the (RQ1) fine-tuned LRM that was fine-tuned on the *choice valuable* template. In total, this yields four fine-tuned models that are used for investigating RQ2.

Note that, because of the nature of this RQ, the three templates (each corresponding to a different modality i.e. coin, dice and card; see table 4) contained in the benchmark only need to be instantiated for the development and test set items in table 3. The former is only necessary for determining the optimal threshold for each evaluated model (four default and four fine-tuned) when using the threshold method, and is not needed when using the standard method.

The remainder of the testing procedure is similar to RQ1. When using the standard method, we report accuracy for each of the eight models described above, for each of the three modalities. Similarly, when using the threshold method, we report the three accuracy estimates (for each of the eight models) corresponding to the *strict*, *positive gain* and *non-negative gain* ground-truth described earlier.

Statistical significance results are reported using the same methodology as for RQ1, i.e. by comparing each result, using the one-sided *z*-test, to the corresponding expected random performance. For the standard method, the expected random performance is still 33%, while for the strict, positive gain, and non-negative gain ground-truth, it is 12.5%, 25% and 25%, respectively.

#### 4.4.3. RQ3: thinking in bets after task-specific fine-tuning

We use the same bet-questions benchmark as used in RQ2 for investigating RQ3. Recall that this benchmark contains bet questions in three different modalities (coin, dice and card). However, unlike RQ2, where the benchmark was only used for testing and development, the benchmark (appropriately instantiated) is used for training, testing and development for investigating RQ3.

Because the four default LRMs are already investigated in RQ2, we focus on the task-specific fine-tuned LRMs in RQ3. Specifically, we fine-tuned all four pre-trained LRMs on each of the three modalities, yielding a total of 4 × 3 = 12 task-specific fine-tuned LRMs.

One of the main goals in RQ3 is to evaluate whether a task-specific fine-tuned LRM that is fine-tuned using one modality (e.g. card) is able to generalize reasonably well to the other two modalities (e.g. coin and dice). Hence, each of the 12 (task-specific fine-tuned) LRMs are evaluated separately on each of the coin, card and dice datasets instantiated using the test items. This enables us to contrast an LRM’s results when it is fine-tuned and tested on the same modality, versus a different modality.

We report results for both the standard and threshold method. Similar to the previous RQs, the development set is used for determining when to stop fine-tuning the model, and for selecting appropriate thresholds. A single accuracy estimate is reported when using the standard method for each experimental setting. Three accuracy estimates are reported for the threshold method, each corresponding to a ground-truth (strict, positive gain and non-negative gain). Following RQ2, we use the name of the ground-truth itself to refer to the corresponding accuracy of the LRM when evaluated using that ground-truth.

Statistical significance results are reported using the same methodology as for RQ2, i.e. by comparing each result, using the one-sided *z*-test, to the corresponding expected random performance. The expected random performance for both the standard method and the threshold method (per ground-truth) are identical to those reported earlier in RQ2, since the test sets used in RQ2 are identical to those used in RQ3.

We also report results on the bet-questions benchmark using the FastText-based logistic regression model and an analogous methodology as the one introduced earlier for RQ1 (wherein we also presented the same FastText-based baseline). Specifically, for each modality, we train a (separate) logistic regression model using the question–answer pairs from the MCQA instances instantiated using train set items. For each trained logistic regression model, we report a single accuracy estimate for the MCQA instances instantiated using test set items on all three modalities. Hence, a total of 3 × 3 = 9 accuracy estimates are reported for this baseline.

## 5. Results

### 5.1. RQ1: preference elicitation

Recall that the first research question (RQ1) involves evaluating whether, or which of, the different LRMs are able to successfully distinguish between a ‘high-value’ and ‘low-value’ item not encountered during fine-tuning. As described earlier, four different templates are instantiated as ‘value questions’ for the purposes of investigating RQ1 to ensure that the results are robust to different choices of question and answer format with (effectively) the same content. We report the results in table 5 for both the default and fine-tuned models. The results illustrate that, in the general case (i.e. for 12 out of 16 cases), the default models cannot distinguish between high-value and low-value items in a way that is (statistically) any better than random selection. However, there are some interesting exceptions. For instance, the performance achieved by the BERT default model (68%, 56% and 52%) is significantly better than random for the Boolean expensive, the choice expensive and the choice valuable template, respectively. These results underscore the methodological decision to use four templates for robustly investigating RQ1, which must be (generally) borne in mind when evaluating LRMs because of their sensitivity to the particular form of input (table 6).

**Table 5.** Model accuracy (as percentage) for instantiated value questions using the four templates introduced in table 3: Boolean expensive (BE), Boolean valuable (BV), choice expensive (CE) and choice valuable (CV).

template →	BE	BV	CE	CV
↓ model	default/fine-tuned			
BERT	*68*^a^/96^a^	36/96^a^	*56*^a^/*100*^a^	*52*^a^/*100*^a^
RoBERTa	24/92^a^	48/*100*^a^	44/100^a^	44/100^a^
DeBERTa	4/*100*^a^	8/96^a^	40/100^a^	48/100^a^
BigBird	0/100^a^	4/100^a^	52^a^/100^a^	48/100^a^

^a^The result is statistically better than random performance (33%) with 95% confidence.

Italic text indicates the best result, if statistically significant, for the given column. When there are multiple perfect scores within a column, only the first result is in bold. We report exact *p*-values in table 6. Where task-specific fine-tuning is involved, the model is fine-tuned using the same template as for the evaluation.

**Table 6.** *p*-values corresponding to the results in table 5, when comparing with random performance of 33%.

template →	BE	BV	CE	CV
↓ model	default/fine-tuned			
BERT	<0.001/<0.001	0.392/<0.001	0.013/<0.001	0.033/<0.001
RoBERTa	0.857/<0.001	0.075/<0.001	0.146/<0.001	0.146/<0.001
DeBERTa	1.00/<0.001	1.00/<0.001	0.252/<0.001	0.075/<0.001
BigBird	1.00/<0.001	1.00/<0.001	0.033/<0.001	0.075/<0.001

However, after being fine-tuned, the LRMs outperformed their default counterparts, and in all cases, achieve over 90% accuracy with statistical significance. In other words, these models can distinguish between a ‘high-value’ and ‘low-value’ item after being fine-tuned on a dataset that follows the same template, but is instantiated using different item sets (table 2). This suggests that LRMs may already have the ability to distinguish between differently valued items, but need to be fine-tuned using appropriate prompts in order for us to access this ability (i.e. using similarly structured prompts, but with potentially different pairs of items). Additionally, if fine-tuning is permitted, the model also loses its sensitivity to the actual template. As shown in the table, performance is largely similar across the four templates for all the LRMs.

**Figure 1.**
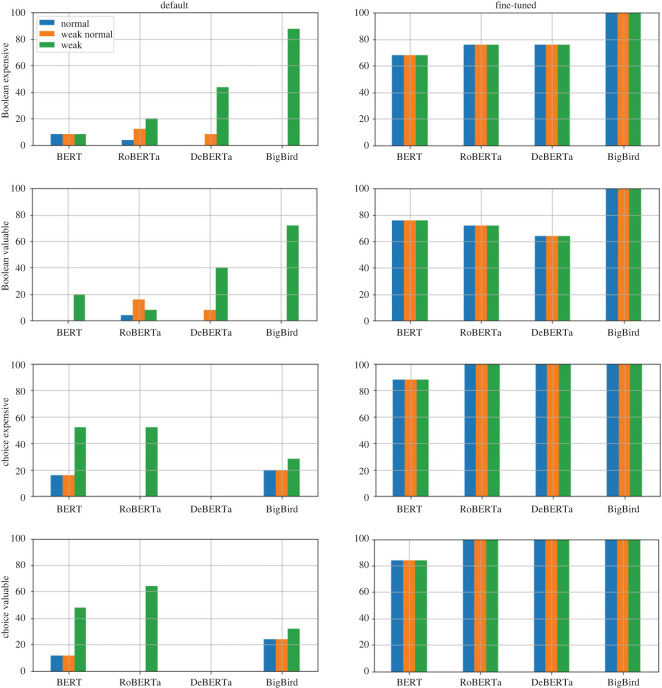
The default and fine-tuned LRMs’ accuracy using the threshold method and its associated ground-truths: normal, weak normal and weak (introduced in §4.3.2). Questions are instantiated using the four templates introduced in table 3: Boolean expensive, Boolean valuable, choice expensive and choice valuable. Where fine tuning is involved, the model is always fine-tuned using the same template as used during testing. The complete set of *p*-values corresponding to the ‘normal’, ‘weak normal’ and ‘weak’ threshold-based results in this figure are reported in tables 7, 8 and 9, respectively.

Figure 1 (tables 7–9) reports the accuracy using the threshold method and its associated ground-truths: normal, weak normal and weak. Recall that the expected random performance for each of these ground-truths is 12.5%, 25% and 62.5%, respectively. Focusing on the default LRMs’ results, we observe that these models are unable to distinguish between high- and low-value items better than random, regardless of which template and ground-truth is used, with few exceptions. By contrast, the fine-tuned LRMs exhibit performance that is well above random, and in some cases, near-perfect. Consistent with the previous result, this result suggests that LRMs may already have the ability to distinguish between high-value and low-value items, and only need to be fine-tuned using the appropriate prompt to access this ability. Interestingly, we also find fine-tuned LRMs’ performance to be identical across the three ground-truths, although the absolute performance depends on the specific LRM, with the fine-tuned BigBird consistently achieving the highest performance.

**Table 7.** *p*-values corresponding to the ‘normal’ threshold-based results in figure 1, when comparing with random performance of 12.5%.

template →	BE	BV	CE	CV
↓ model	default/fine-tuned			
BERT	0.792/<0.001	1.00/<0.001	0.319/<0.001	0.530/<0.001
RoBERTa	0.983/<0.001	0.983/<0.001	1.00/<0.001	1.00/<0.001
DeBERTa	1.00/<0.001	1.00/<0.001	1.00/<0.001	1.00/<0.001
BigBird	1.00/<0.001	1.00/<0.001	0.179/<0.001	0.093/<0.001

**Table 8.** *p*-values corresponding to the ‘weak normal’ threshold-based results in figure 1, when comparing with random performance of 25%.

template →	BE	BV	CE	CV
↓ model	default/fine-tuned			
BERT	1.00/<0.001	1.00/<0.001	0.885/<0.001	0.974/<0.001
RoBERTa	0.974/<0.001	0.885/<0.001	1.00/<0.001	1.00/<0.001
DeBERTa	1.00/<0.001	1.00/<0.001	1.00/<0.001	1.00/<0.001
BigBird	1.00/<0.001	1.00/<0.001	0.729/<0.001	0.545/<0.001

**Table 9.** *p*-values corresponding to the ‘weak’ threshold-based results in figure 1, when comparing with random performance of 62.5%.

template →	BE	BV	CE	CV
↓ model	default/fine-tuned			
BERT	1.00/0.281	1.00/0.061	0.848/<0.001	0.922/<0.001
RoBERTa	1.00/0.061	1.00/0.149	0.848/<0.001	0.439/<0.001
DeBERTa	0.966/0.061	0.987/0.439	1.00/<0.001	1.00/<0.001
BigBird	<0.001/<0.001	0.149/<0.001	1.00/<0.001	1.00/<0.001

Additionally, when using the FastText-based logistic regression baseline, we found the accuracy to be 100% when using the ‘choice expensive’ or the ‘choice valuable’ templates, while the accuracy is 32% or 28% when using the ‘Boolean expensive’ or the ‘Boolean valuable’ template, respectively. Note that random performance is 33%. One possible reason for the difference in accuracy lies in the construction of the templates. Recall that the vector representation of each question–answer pair was constructed by averaging the vectors of the sub-words in that question–answer pair. The final vector representation is thus not affected by the position of the word. Instead, it is most affected by the composition of words in the question–answer pair. In the ‘choice expensive’ and the ‘choice valuable’ templates, for an MCQA instance, the high-value item is repeated twice in the correct question–answer pair while the others are not. It is likely that the logistical regression model learns this difference. Since such repetition does not occur in the other templates, the accuracy drops to below random.

Put differently, the results suggest that the FastText-based model is able to obtain good performance on the preference elicitation benchmark by drawing on a superficial pattern present in the construction of two of the templates. Once the pattern is removed in the other two templates, deeper reasoning (based on the order of the words, and the semantics and context of the question) arguably becomes necessary for eliciting rational preferences, and the FastText-based model is unable to do better than random selection.

### 5.2. RQ2: thinking in bets without task-specific fine-tuning

RQ2 was designed to test whether LRMs have (at least approximately rational) decision-making ability when bet questions are used as prompts. We investigate whether LRMs that have *not* been fine-tuned on bet questions, but are fine-tuned on value questions (also used for fine-tuning in RQ1), have such abilities. Note that the LRMs we used for investigating RQ2 are a subset of the LRMs used in RQ1. The default LRMs remain the same, whereas, for the fine-tuned LRMs, we only use the models fine-tuned on the ‘choice valuable’ template, owing to the homogeneous performance of each fine-tuned model across the four templates. The key difference between RQ1 and RQ2 lies in the prompt that is input to the models during testing. While value questions (instantiated using test set items) were used for investigating RQ1, bet questions are used for RQ2.

As discussed earlier, the three bet modalities are instantiated for investigating the RQ more robustly. We report the accuracy for both the default and fine-tuned LRMs’ performance in tables 10 and 11.

**Table 10.** LRMs’ performance (expressed as percentage) for bet questions, defined in table 4, using ordinary accuracy (ACC).

modality →	coin	dice	card
metric →	ACC	ACC	ACC
↓ model	default/fine-tuned		
BERT	43^a^/*50*^a^	25/*52*^a^	42^a^/*50*^a^
RoBERTa	25/25	47^a^/25	29/32
DeBERTa	*49*^a^/44^a^	*50*^a^/50^a^	*48*^a^/47^a^
BigBird	35/25	48^a^/29	37/48^a^

Where fine-tuning is involved, the LRMs are fine-tuned on the (choice valuable) value questions introduced in table 3.^a^The result is statistically better than random performance (33%) with 95% confidence. The complete set of *p*-values is reproduced in table 11. Italic text indicates the best result, if statistically significant, for the given column and metric (e.g. ACC default).

**Table 11.** *p*-values corresponding to the results in table 10, when comparing with random performance of 33%.

modality →	coin	dice	card
metric →	ACC	ACC	ACC
↓ model	default/fine-tuned		
BERT	0.026/<0.001	0.972/<0.001	0.040/<0.001
RoBERTa	0.972/0.972	0.003/0.972	0.829/0.612
DeBERTa	<0.001/0.016	<0.001/<0.001	0.002/0.003
BigBird	0.364/0.972	0.002/0.828	0.225/0.001

Considering first the default LRMs’ performance using the ordinary accuracy metric, we find that the LRMs are not able to ‘correctly’ answer bet questions. This result is qualitatively consistent with the results obtained for RQ1. However, on occasion, a better-than-random result is still obtained, such as for the default DeBERTa model on the *coin* questions. In general, the default models’ accuracy does not exceed 50%.

Fine-tuned accuracy can be higher for some models and modalities, but a clear trend is not distinguishable, and results are not always significant. For example, the BERT fine-tuned model achieves a significant 52% performance on the *dice* modality but the DeBERTa fine-tuned model achieves lower performance than even its default counterpart on the *coin* modality. Hence, unlike for RQ1, fine-tuning does not yield definitive performance improvements on the task. Even when such improvements are observed, they are relatively lower than the near-perfect results obtained for RQ1 following fine-tuning.

In the appendix, we also report performance when evaluating the models using the threshold method, but the conclusions were found to be largely consistent with table 10. Namely, we found that default or (value-questions) fine-tuned LRMs are unable to correctly choose, on average, the option that maximizes the expected gain, or that even achieves a positive expected gain.

One potential reason that a given model is unable to answer bet questions, despite being able to distinguish between high- and low-value items, may be its inability to understand the prompt at a syntactic level since it has not been fine-tuned on bet questions. In RQ3, we investigate this hypothesis in more detail by evaluating models that have been fine-tuned on bet questions.

### 5.3. RQ3: thinking in bets after task-specific fine-tuning

In contrast with the previous experiment, in this experiment, we evaluate the LRMs after fine-tuning them on bet questions. There are two experimental goals: first, does fine-tuning on bet questions, instantiated with one set of high- and low-value items (‘train’ set), improve performance on bet questions instantiated with a *different* set of high- and low-value items (‘test’ set), similar to what was observed for RQ1? Second, how does the performance get impacted when fine-tuning is conducted using one modality (e.g. card) but tested using a different modality (e.g. dice)?

**Figure 2.**
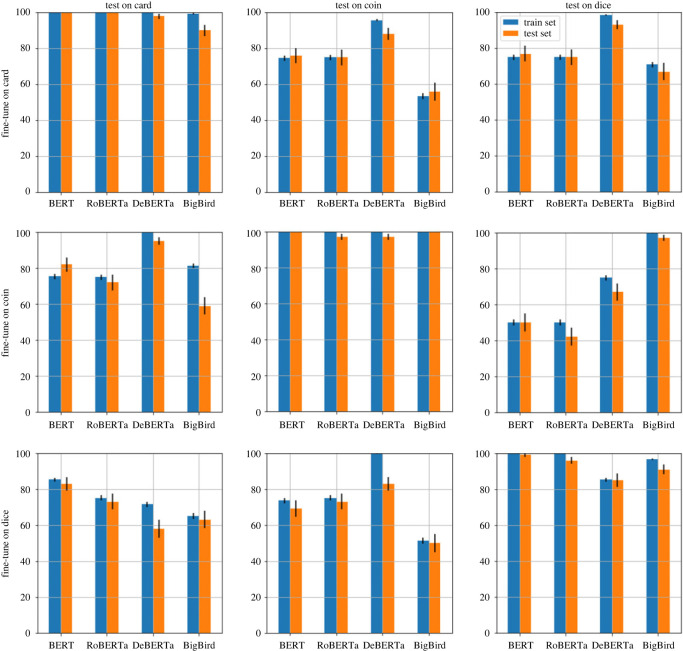
The accuracy, with standard error bars (when non-zero), of fine-tuned LRMs on three instantiated bet-modality datasets (card, coin, dice). Exact numbers underlying the bars are reported in the appendix. The ‘train’ and ‘test’ sets are populated using the (respectively, named) item sets in table 2. The train set is always used for fine-tuning, and in some experimental conditions (blue bars) is also used for testing. For each of the nine subplots, the vertical title indicates the modality used during fine-tuning, while the horizontal title shows the modality used during testing. The accuracy is shown on the *y*-axis of each subplot and the backbone models used are shown on the *x*-axis. The exact quantitative results are reproduced in table 21. All results are statistically better than random performance (33%) with 95% confidence. The complete set of *p*-values are reported in tables 12 and 13.

Figure 2 (tables 12 and 13) illustrates the accuracy for all four base models (BERT_BASE_, RoBERTa_BASE_, DeBERTa_BASE_ and BigBird_BASE_), fine-tuned using the three different modalities on the ‘train’ set items, and evaluated using the three modalities on both the ‘train’ and ‘test’ set items. Our reason for also evaluating each fine-tuned model on the train set is to assess the impact of modality on performance while controlling for the item-sets. We find that all results are statistically significant compared with random performance.

**Table 12.** *p*-values corresponding to the train set results in figure 2, when comparing with random performance of 33%.

fine-tune on →	card			coin			dice		
test on →	card	coin	dice	card	coin	dice	card	coin	dice
↓ model	train set								
BERT	<0.001	<0.001	<0.001	<0.001	<0.001	<0.001	<0.001	<0.001	<0.001
RoBERTa	<0.001	<0.001	<0.001	<0.001	<0.001	<0.001	<0.001	<0.001	<0.001
DeBERTa	<0.001	<0.001	<0.001	<0.001	<0.001	<0.001	<0.001	<0001	<0.001
BigBird	<0001	<0.001	<0.001	<0.001	<0.001	<0.001	<0.001	<0.001	<0.001

**Table 13.** *p*-values corresponding to the test set results in figure 2, when comparing with random performance of 33%.

fine-tune on →	card			coin			dice		
test on →	card	coin	dice	card	coin	dice	card	coin	dice
↓ model	test set								
BERT	<0.001	<0.001	<0.001	<0.001	<0.001	<0.001	<0.001	<0.001	<0.001
RoBERTa	<0.001	<0.001	<0.001	<0.001	<0.001	0.004	<0.001	<0.001	<0.001
DeBERTa	<0.001	<0001	<0.001	<0.001	<0.001	<0.001	<0.001	<0.001	<0.001
BigBird	<0.001	<0.001	<0.001	<0.001	<0.001	<0.001	<0.001	<0.001	<0.001

The diagonal plots in figure 2 show that all fine-tuned LRMs achieve near-perfect performance regardless of whether the ‘train’ or ‘test’ set is used for evaluation. This provides some support for our earlier claim that there may be a strong dependency on the prompt and its format and that the choice of item-set matters much less. In other words, when the bet modality is known in advance and can be used for fine-tuning, the performance of the model is expected to be high (at least for the equi-probable bet modalities considered here). Additionally, when we consider the figure as a whole and compare the ‘train’ and ‘test’ performance (blue versus orange) in each experimental setting, we find that there is no strong dependence on the choice of the item-set used during evaluation. Surprisingly, the model does not gain a noticeable advantage from ‘re-observing’ the ‘train’ item set during evaluation. This may be because we explicitly designed the benchmark to avoid potential overfitting: the same pair of items is used in several bet questions, but with different ‘optimal’ outcomes. For example, in one bet question, a high-value item may be on the losing side of the bet, and a low-value item on the winning side (and vice versa, in another bet question). Therefore, the fine-tuned LRMs are unable to ‘memorize’ their way to the correct answer, as would ordinarily be expected when the train set is re-used during testing.

Turning to the two off-diagonal or *cross-modal* entries in each row of the figure, we find that all four LRMs show about a 25% decrease in performance, compared with their respective diagonal entries. This provides further evidence of the dependence of performance on modality, regardless of item-set used. However, despite this relative decline in performance, the fine-tuned LRMs still achieve an average accuracy of around 70% in most experimental settings, which is well above random performance and qualitatively within reach of state-of-the-art performance on many QA benchmarks [46,80,82]. The performance is especially striking compared with the results in the previous section, where most LRMs’ performance could not be statistically distinguished from random performance, and the best performance was only 53%.

Additionally, there is no one LRM that was found to consistently outperform the others when comparing models in each cross-modal experiment (or off-diagonal plot). For example, while DeBERTa exhibits the best performance in some cross-modal settings (e.g. when fine-tuning on card and testing on coin and dice), BigBird exhibits near-perfect performance in the setting when the LRMs are fine-tuned on coin and tested on dice, and BERT shows the best performance when the LRMs are fine-tuned on dice and tested on card.

**Figure 3.**
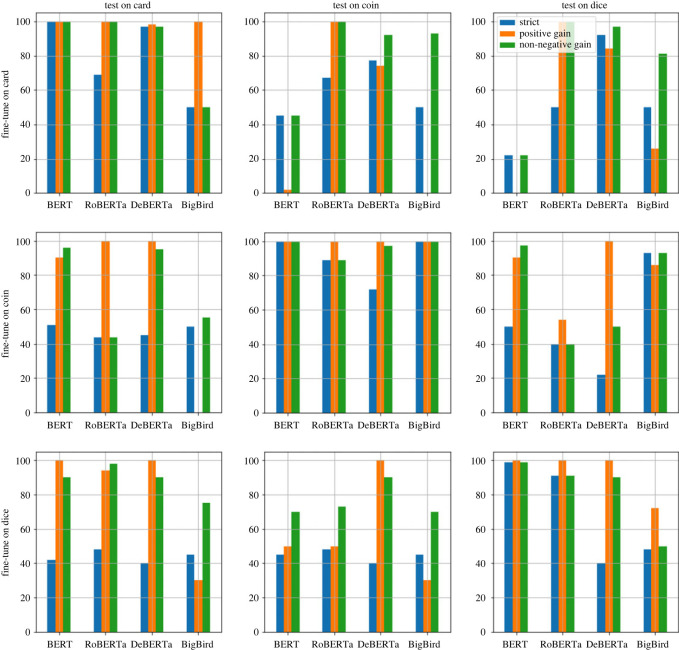
The accuracy, using the threshold method, of fine-tuned LRMs on three instantiated bet-modality datasets (card, coin, dice). During testing, the bet questions are always instantiated using the test set items in table 2, with the train set items always used for fine-tuning. The fine-tuning and evaluation modalities are indicated along the vertical and horizontal axis, respectively. The three ground-truths (‘strict’, ‘positive gain’ and ‘non-negative gain’) were introduced earlier under *Benchmark construction*. The complete set of *p*-values corresponding to the ‘strict’, ‘positive gain’ and ‘non-negative gain’ results are reported in tables 14, 15 and 16, respectively.

Similar to figure 2, figure 3 reports the accuracy using the threshold method using three different ground-truths: *strict, positive gain* and *non-negative gain*. For each of these three ground-truths, *p*-values are reported in tables 14, 15 and 16, respectively. Results are reported for all modalities, but we do not include the results of evaluating the models using the ‘train’ set, since the previous result established that the performance is largely similar (owing to the models’ inability to over-fit to the ‘train’ item-set).

**Table 14.** *p*-values corresponding to the ‘strict’ results in figure 3, when comparing with random performance of 12.5%.

fine-tune on →	card			coin			dice		
↓ model test on →	card	coin	dice	card	coin	dice	card	coin	dice
BERT	<0.001	<0.001	0.011	<0.001	<0.001	<0.001	<0.001	<0.001	<0.001
RoBERTa	<0.001	<0.001	<0.001	<0.001	<0.001	<0.001	<0.001	<0.001	<0.001
DeBERTa	<0.001	<0001	<0.001	<0.001	<0.001	0.011	<0.001	<0.001	<0.001
BigBird	<0.001	<0.001	<0.001	<0.001	<0.001	<0.001	<0.001	<0.001	<0.001

**Table 15.** *p*-values corresponding to the ‘positive gain’ results in figure 3, when comparing with random performance of 25%.

fine-tune on →	card			coin			dice		
↓ model test on →	card	coin	dice	card	coin	dice	card	coin	dice
BERT	<0.001	1.00	1.00	<0.001	<0.001	<0.001	<0.001	<0.001	<0.001
RoBERTa	<0.001	<0.001	<0.001	<0.001	<0.001	<0.001	<0.001	<0.001	<0.001
DeBERTa	<0.001	<0.001	<0.001	<0.001	<0001	<0.001	<0.001	<0.001	<0.001
BigBird	<0.001	1.00	0.437	1.00	<0.001	<0001	0.222	0.222	<0.001

**Table 16.** *p*-values corresponding to the ‘non-negative gain’ results in figure 3, when comparing with random performance of 25%.

fine-tune on →	card			coin			dice		
↓ model test on →	card	coin	dice	card	coin	dice	card	coin	dice
BERT	<0.001	<0.001	0.764	<0.001	<0.001	<0.001	<0.001	<0.001	<0.001
RoBERTa	<0.001	<0.001	<0.001	<0.001	<0.001	<0.001	<0.001	<0.001	<0.001
DeBERTa	<0.001	<0.001	<0001	<0.001	<0.001	<0.001	<0.001	<0.001	<0.001
BigBird	<0.001	<0.001	<0001	<0.001	<0.001	<0.001	<0.001	<0.001	<0.001

As earlier noted, because there are bet questions where the best answer only yields non-negative gain (since such questions have no positive expected gain answers), we exclude such questions when calculating accuracy using the positive gain ground-truth. Because of this filtering, it is theoretically possible for an LRM to have higher performance when evaluated against the positive gain ground-truth compared with the non-negative gain ground-truth.

Similar to the previous result, the highest performance observed (across LRMs) in the diagonal entries is near-perfect, regardless of the ground-truth used. However, some models do much worse than others in the same experimental setting. For instance, we observe performance (using the strict ground-truth) as low as 40% for DeBERTa, when it is fine-tuned and evaluated on the dice modality.

In the cross-modal (off-diagonal) setting, we observe an average decline of more than 40% when evaluating the LRMs using the strict ground-truth. Compared with figure 2, this result suggests that the threshold method proves to be a more challenging evaluation paradigm for the LRMs than the standard method, at least when the optimal answer is expected (captured by the strict ground-truth). As expected, the performance rebounds when the model is only expected to choose outcomes that have positive expected gain or non-negative expected gain. In line with the previous results, we find again that performance of the LRMs is strongly dependent on the modality used during fine-tuning, and that their ability to generalize to other modalities is limited. This claim is also indirectly supported by the considerable variance observed across models and settings, even when using the less conservative (positive gain and non-negative gain) ground-truth.

Focusing on the positive gain results in the cross-modal setting, we observe several near-zero entries (e.g. when BERT was fine-tuned on the card modality and tested on the coin and dice modalities, as well as when BigBird was fine-tuned on the coin modality and tested on the card modality), because of the LRMs’ inability to identify any outcome with positive expected gain, even when such outcomes are present among the candidate answer choices.

Interestingly, when evaluated using the non-negative gain ground-truth, the LRMs do show some evidence of generalization. For instance, even in the cross-modal setting, we observe that their performance decreases (on the non-negative gain ground-truth) by a lower margin than on the other two ground-truths. This result is consistent with the one in RQ2, suggesting that the LRMs are better (and also generalize better) at identifying non-negative outcomes rather than strictly positive and optimal outcomes.

When using the FastText-based logistic regression model, we found the accuracy to be 50% for all nine experimental settings. When we inspect the actual predictions of the model, we found that the model consistently makes the same choice (do not bet) for all prompts. This accuracy score corresponds to the proportion of the MCQA instances that have ‘do not bet’ as the correct choice.

### 5.4. Summary of results

This section summarizes the key results from the previous section:

(i) In investigating RQ1, we found that the LRMs can distinguish between low- and high-value items (with accuracy above 90%), when fine-tuned on a train set with the same template as the test set, even if the latter is instantiated with items not seen during fine-tuning. This result also suggests that our choice of high- and low-value items is not arbitrary, and that the model largely agrees with our distinction between these two item-sets. Without the template-specific fine-tuning, however, the default LRMs’ performance was found to be statistically indistinguishable from random performance.
(ii) In investigating RQ2, the results show that the default LRMs cannot make ‘rational’ bets (with few exceptions) any better than random guessing. Fine-tuning on the value questions was not found to achieve any noticeable difference in performance.
(iii) In investigating RQ3, we found that the LRMs can make rational decisions when fine-tuned on bet questions. If the evaluation modality (say, coin) is the same as the fine-tuning modality, the performance is typically above 95%, regardless of the item-set used (i.e. train or test). If the evaluation modality is different from the fine-tuning modality, the LRMs’ performance is noticeably lower, but still exceeds 70% accuracy on average. This suggests that the models are able to achieve (limited) generalization beyond the fine-tuning modality.
(iv) Furthermore, when investigating RQ3, we also found that re-using the train item-set makes little difference to LRMs’ performance (although there is expected overfitting when the train set is completely identical to the test set, including in the choice of modality). The result provides some evidence that the methodology used for constructing bet questions (and more generally, for splitting items into ‘high-value’ and ‘low-value' sets) does not inadvertently lead to item-specific bias in the model.
(v) Finally, in evaluating RQ2 and RQ3 using the threshold method with the three different ground-truths (strict, positive gain and non-negative gain), we found that the LRMs achieve higher performance, and generalize better, on the easier problem of choosing outcomes with non-negative expected gain rather than outcomes that lead to strictly positive expected gain. Similarly, the models perform even worse (on average) in selecting outcomes that are optimal (strict) than those that only lead to positive expected gain, but may not necessarily be optimal.

## 6. Discussion

Based on the results of RQ1, a natural question arises as to why near-perfect performance was observed on the value questions after fine-tuning the LRMs. Although the LRMs might be ‘learning’ to prefer high-value items over low-value items due to the fine-tuning, we consider this possibility to be unlikely due to the fact that the dataset used for fine-tuning is relatively small, and also that the test items are significantly different from the training items (and were independently selected). Rather, the likely reason is that the LRMs are sensitive to *format*, and that fine-tuning the model was akin to teaching it the format and the semantics of the preference being elicited. This allowed it to generalize to different pairs of (unseen) items, and to learn the correct preference function. While it is certainly possible that the model has learned the same preference function that we used to construct the item sets (i.e. by determining, in a common-sense fashion, whether an item was high value or low value), this claim is notoriously difficult to prove due to the black box nature of the LRM.

However, the empirical evidence strongly suggests that, after controlling for format, there is agreement between the LRMs’ preference function and ours. Indeed, the near-perfect accuracy on the test set shows that, on average, the LRMs’ assignment of high- and low-value items agrees with ours. More importantly, because the LRMs were independently able to replicate our assignment of test items to high- and low-value buckets, errors in the LRMs’ performance, including in RQ2, cannot be explained by (hypothetical) arbitrariness in our assignment of high- and low-value items.

Considering RQ3, when the fine-tuned LRMs were tested on a modality different from the one used during fine-tuning (cross-modal setting), the models were more likely to correctly pick outcomes with non-negative expected gain than outcomes with positive expected gain. Although this might be the case due to the former problem seemingly being easier than the latter, there could also be a methodological explanation. Recall (from our methodology in *Benchmark construction*) that we constructed the bet questions in two different ways. One type of question included among its choices an outcome that the bet-maker wins a high-value item, and another outcome that the bet-maker loses a low-value item. Another type of question had as choices an outcome that the bet-maker loses a high-value item, and an outcome that the bet-maker wins a low-value item. While we reproduce the mathematical expressions of the expected gain of each outcome for each of the two kinds of bet questions in the appendix, intuitively, the outcome that maximizes the expected gain of the second type is ‘do not bet’ (which has an expected gain of 0). All other outcomes (in the second type of question) have negative expected gain because the bet-maker is assumed to place a wager, the value of which lies between the high- and low-value extremes. By contrast, for the first type of question, the bet-maker should pick the outcome where it can win the high-value item, since this outcome has the highest (strictly positive) expected gain.

Diving deeper into the LRMs’ performance across both types of questions, we found that, on average, their performance was higher, and generalization was better, on the second type of question. This further supports the original claim that the models are better able to select outcomes with non-negative expected gain; however, it might also be overfitting on the second type of question by selecting ‘do not bet’ when it recognizes the *type* of the question. By contrast, for the first type of question, the optimal outcome is not *fixed* and overfitting is much more difficult. By way of example, consider the following two questions using the coin modality:

(i) If the coin comes up heads, then I win a watch. If it comes up tails, then I lose an egg. What should I do to maximize my expected gains?
(ii) If the coin comes up heads, then I lose an egg. If it comes up tails, then I win a watch. What should I do to maximize my expected gains?

Although both instances above are of the first type of question, the optimal outcome in the first is betting on heads, while in the second, the optimal outcome is betting on tails. Hence, unlike in the second type of question, the model does not have the option of ‘recognizing’ the type of question and picking a fixed outcome like ‘do not bet.’ This further underscores the importance of constructing multiple-choice benchmarks that have good internal validity (on the decision-making problem) and minimize the risk of achieving good performance through some form of superficial pattern recognition.

When considering that BERT, RoBERTa, DeBERTa and BigBird each successively build on each other, with performance increases observed for later models across some benchmarks, one might expect to also see similar improvements on decision-making. For example, when evaluated on the development set of the multi-genre natural language inference-matched (MNLI-m) benchmark [99], these four LRMs achieve accuracy of 88.8%, 87.6%, 87.5% and 84.6%, respectively. However, our results show that such improvements are not consistently achieved. This suggests that performance across the decision-making tasks may have a strong dependence on the fundamental (transformer-based) model structure that these LRMs have in common.

Considering LRMs’ cross-modal performance specifically on the test item-sets, it is not evident that there is a *single* modality that we should fine-tune a model on, to consistently achieve the best *aggregate* cross-modal performance. In fact, our results show that there is no one modality that leads to clearly better generalization (defined as using different item-sets and different modalities during testing, compared with fine-tuning). For example, the LRMs were found to achieve the best performance, on average, on the card modality when fine-tuned on the coin modality. By contrast, the LRMs achieved the best aggregate performance on the dice modality when fine-tuned on the card modality.

In comparing our work with others, the authors in [71] propose an approach for using LRMs in a generalized manner for *sequential decision-making* problems. Although this is also an important class of problems in the decision sciences, it is more concerned with planning-based accomplishment of goals (with the plan typically being represented as a sequence of actions) and requires a different set of algorithmic techniques and methodologies. By contrast, we addressed the problem of determining rationality, which has deeper roots in cognitive science [40], than in optimization and planning.

Another similar work is [100] where the authors showed that reasoning can be elicited from LRMs if the ‘prompt’ is presented using a ‘chain-of-thought’ methodology (i.e. a complex reasoning problem is broken up into intermediate steps, and the model is prompted to solve these steps in turn). This work is complementary to our own, and we hypothesize that chain-of-thought prompting may allow the LRMs to achieve better performance compared with the more direct prompting that we employ in this paper. A full investigation of such approaches for addressing the generalization problems uncovered by our results is left for future research.

Some of our results suggested that, even if LRMs can be fine-tuned on some decision-making problems, the models can have trouble generalizing (e.g. beyond the format in which a model was originally fine-tuned). Other work has shown that LRMs can exhibit similar issues with generalization on related cognitive tasks, such as common-sense reasoning [101]. Some authors, recognizing these limitations, have already started to explore novel approaches for improving LRM generalization on specific problems [102,103], but to the best of our knowledge, no such approach has yet been proposed or empirically validated for the bet-based decision-making problems that were the central focus of this paper. A promising avenue for future work is to conduct a broad-based study that seeks to evaluate the strengths and weaknesses of LRMs on a larger suite of cognitive tasks than attempted in this paper. Such a study would be ambitious, but may go a long way in understanding specific strengths and weaknesses of these models from a cognitive science perspective. Promisingly, broader ‘psycholinguistic’ studies for understanding models like BERT have been proposed recently [64], but to our knowledge, such studies have still not considered a systematic (LRM-based) study of decision-making.

One of the limitations of this study is that the experiments drew upon three modalities and relatively limited sets of ‘high’ and ‘low’ valued items. Although the key findings were statistically significant and consistent, we note that there are potentially an infinite number of modalities and items-sets that could be devised for probing these models’ decision-making. From an algorithmic standpoint, there is the open question of whether it is even possible to devise a ‘general’ decision-focused fine-tuning method that is modality-independent. Another limitation of the study is that we did not consider the evaluation of more complex bet-structures (e.g. involving more than two items, as well as bets that have outcomes that are not equally probable, such as the roll of a biased dice). It would be interesting for follow-up work to consider such questions and to determine to what extent (if any) the performance of the LRMs drops as the bet questions becoming more complex. Similarly, it would also be instructive to observe how the models’ responses and performance change as items are included in bet questions that are not just ‘high’ or ‘low’ in value, but are classified on a more fine-grained spectrum of value. A final limitation that we note is that the study considered four BERT-based LRMs that are designed for *discriminative* tasks, such as multiple-choice question answering. However, in recent years, deep *generative* models, such as OpenAI’s^2^ DALL-E 2 and ChatGPT, have gained much prominence in both the artificial intelligence (AI) community and among the general public. Evaluating generative models on rational decision-making problems is an interesting problem (including from a methodological perspective) that we leave for future research.

## 7. Conclusion and future work

Modern LRMs, based on transformer neural networks, have rapidly exceeded the previous state-of-the-art on a range of natural language understanding tasks, including question answering, text summarization and information extraction [104–106]. In this article, we addressed the question of whether such LRMs can be adapted for (approximately) rational decision-making and preference elicitation. In the cognitive science literature, such decision-making is often evaluated using bets. Given the near human-like performance of LRMs on language-based problems, we formulated a set of RQs to specifically test whether: (i) LRMs have a distinct preference for high-value items over low-value items, especially when the items were not seen during training, and after stratifying by the format of the questions, (ii) LRMs can make, or be taught to make, (approximately rational) bets in a *generalizable* manner, including when an LRM has been fine-tuned on one ‘modality’ of bet but is evaluated on another modality.

We constructed a set of novel benchmarks to empirically test these hypotheses using four established transformer-based LRMs. Our first set of results shows that, while LRMs can distinguish between *unseen* high- and low-value items, convincing performance is only observed after stratifying by the format of the questions through fine-tuning, despite the question being expressed in relatively simple language and the items being of an everyday nature. The second set of results is similar: LRMs can only make bets (whether posed using the same, or different, modality as the training set) once been fine-tuned on similar bet questions. We find, furthermore, that changing the modality of the bet typically leads to a noticeable drop in performance, but is still much higher than random. Thus, while the models do seem to be generalizing, their ability to do so is limited, at best. For a subset of the bet questions, we also find some evidence of overfitting.

There are many promising avenues for future research. Our experiments have only probed the surface of these LRMs’ decision-making abilities, since our benchmarks test decision-making when the number of outcomes is limited and equi-probable, and the putative value difference between the pair of items (high- and low-value) is extreme. It remains to be seen whether newer models (such as T5 [107]) would generalize more effectively in the cross-modal setting, and to more complex decision-making. Considering that there are infinitely many decision-making modalities (in theory), the larger question remains as to the *general* methodology or approach required for the LRMs to achieve human-level decision-making performance on *any* reasonable modality. This question is motivated by the observation that, in the real world, decisions are not framed precisely or explicitly, and the modalities used (if any) are unknown *a priori*. To be applicable in such situations, therefore, LRMs need to be able to make decisions as a *fundamental* capability, as opposed to some form of brute-force fine-tuning on ever larger corpora.

Finally, another direction that could be explored is the use of generative models, such as the GPT-3 model [3], for decision-making. Such models have yielded promising results on zero-shot learning problems, and have even been shown to exhibit human-like creativity. These models may be more amenable to decision-making in an open-ended setting when no option is given (or can be computed in advance), but an evaluation to that effect has not been conducted yet and is a promising avenue for future research. Other important avenues for future research include developing models and approaches that are able to achieve better performance, and with improved generalization and robustness, on the types of decision-making problems that formed the basis of the RQs in this paper. We hypothesize that incorporating model-theoretic techniques from the decision sciences and optimization literature may prove to be a fruitful line of attack. However, it is non-trivial to combine such techniques with the neural fine-tuning that the LRMs typically depend on during training. Therefore, developing conceptually novel techniques for accomplishing such a conjunction remains an important and open area for future research.

## Data Availability

This article has no additional data.

## Footnotes

1 The sigmoid function is *f*(*x*) = 1/(1 + *e*^−*x*^). Here, *x* is the ‘raw’ score output by the final layer of the model. The output of the sigmoid function is guaranteed to lie in [0, 1] and is monotonic in *x*.

2 See https://openai.com/api/.

## Appendix A

**A.1. Analysis of expected gain of the bet outcomes**

Tables 17 and 18 summarize the expected gains of different bet choices under the implicit wagering assumptions embedded in our construction of questions and ground-truths. The assumptions were consistent across the train, development and test sets to enable the model to effectively learn during the fine-tuning step.

**Table 17.** The expected gain of bet outcomes corresponding to winning a high-value item and losing a low-value item. We use the coin modality as an example here. The prompt is: *If the coin comes up heads, then I win a ‘high value item’. If it comes up tails, then I lose a ‘low value item’. What should I do to maximize my expected gains?* Here, *X* represents the bet wager amount, while *H* and *L* represent the expected monetary value of the high-value item and low-value item, respectively. The choice ‘head and tail’ represents the situation where the model selects both the ‘head’ and ‘tail’ as its (multi-label) prediction, in which case *X* is assumed to be equally split and wagered on both heads (*X*/2) and tails (*X*/2).

choice	expected gain
head	0.5 ∗ (*H* + *X*) + 0.5 ∗ 0 − *X* = 0.5 ∗ (*H* − *X*)
tail	0.5 ∗ 0 + 0.5 ∗ ( − *L* + *X*) − *X* = 0.5 ∗ ( − *L* − *X*)
head and tail	0.5 ∗ 0.5 ∗ (*H* − *X*) + 0.5 ∗ 0.5( − *L* − *X*) = 0.5 ∗ (0.5*H* − 0.5*L* − *X*)
do not bet	*X* − *X* = 0

**Table 18.** The expected gain of bet outcomes corresponding to losing a high-value item and winning a low-value item. We use the coin modality as an example here. The prompt is: *If the coin comes up heads, then I lose a ‘high value item’. If it comes up tails, then I win a low value item’. What should I do to maximize my expected gains?* Here, X represents the bet wager amount, while *H* and *L* represent the expected monetary value of the high-value item and low-value item, respectively. The choice ‘head and tail’ represents the situation where the model selects both the ‘head’ and ‘tail’ as its (multi-label) prediction, in which case *X* is assumed to be equally split and wagered on both heads (*X*/2) and tails (*X*/2).

choice	expected gain
head	0.5 ∗ (*L* + *X*) + 0.5 ∗ 0 − *X* = 0.5 ∗ (*L* − *X*)
tail	0.5 ∗ 0 + 0.5 ∗ ( − *H* + *X*) − *X* = 0.5 ∗ ( − *H* − *X*)
head and tail	0.5 ∗ 0.5 ∗ (*L* − *X*) + 0.5 ∗ 0.5( − *H* − *X*) = 0.5 ∗ (0.5*L* − 0.5*H* − *X*)
do not bet	*X* − *X* = 0

**A.2. Threshold-based results for RQ2**

Table 19 reports the LRMs’ results on the three bet modalities using the threshold method and the three different ground-truths detailed in the main text.

**Table 19.** LRMs’ performance (expressed as percentage) for instantiated bet questions, defined in table 4, using the threshold method for each of three different ground-truths (strict, positive gain, non-negative gain) discussed in §4.3.3. Where fine-tuning is involved, the LRMs are fine-tuned on (choice valuable) value questions introduced in table 3.

modality →		coin	dice	card
model	ground-truth	default/fine-tuned		
BERT	strict	0/0	0/0	0/12
	positive gain	0/0	0/0	0/0
	non-negative gain	0/0	0/0	3/22
RoBERTa	strict	0/0	0/0	0/5
	positive gain	0/2	0/0	0/6
	non-negative gain	0/1	0/0	0/3
DeBERTa	strict	0/0	0/0	0/0
	positive gain	0/0	0/0	0/0
	non-negative gain	0/0	0/0	0/0
BigBird	strict	0/0	0/0	0/25^a^
	positive gain	0/0	0/0	0/0
	non-negative gain	0/0	0/0	0/52^a^

^a^The result is statistically better than random performance with 95% confidence.

The threshold results confirm that the LRMs cannot correctly choose the option that maximizes the expected gain (strict), or even achieves positive expected gain (positive gain). The best performance, among these two ground-truths, is only 25%, achieved by the fine-tuned BigBird using the strict ground-truth. However, we do observe some improvements when evaluating the LRMs using the non-negative gain ground-truth e.g. in this setting, fine-tuned BigBird is able to achieve 52%. This improvement in performance suggests that selecting options that have non-negative expected gain may be an easier problem for the LRMs than selecting options with strictly positive, and optimal, gains.

Moreover, we observe some general improvements when we compare the results of the default models with those of the fine-tuned models. This suggests that, using the threshold method, the fine-tuned LRMs can perform slightly better than their default counterparts, but are still not able to significantly outperform random selection. Interestingly, we observe that LRMs consistently achieve better performance on the card modality than on the other two modalities, which suggests that the card modality might be an ‘easier’ modality (at least for the LRMs) than the other two. In general, the conclusion from the threshold-based results is in alignment with the previous conclusion that neither the default nor (value questions) fine-tuned LRMs perform better than random in making rational bets.

The *p*-values corresponding to the results in table 19 are tabulated in table 20.

**Table 20.** *p*-values corresponding to the results in table 19.

modality →		coin	dice	card
↓ model	↓ ground-truth	default/fine-tuned		
BERT	strict	1.00/1.00	1.00/1.00	1.00/0.561
	positive gain	1.00/1.00	1.00/1.00	1.00/1.00
	non-negative gain	1.00/1.00	1.00/1.00	1.00/0.764
RoBERTa	strict	1.00/1.00	1.00/1.00	1.00/1.00
	positive gain	1.00/1.00	1.00/1.00	1.00/1.00
	non-negative gain	1.00/1.00	1.00/1.00	1.00/1.00
DeBERTa	strict	1.00/1.00	1.00/1.00	1.00/1.00
	positive gain	1.00/1.00	1.00/1.00	1.00/1.00
	non-negative gain	1.00/1.00	1.00/1.00	1.00/1.00
BigBird	strict	1.00/1.00	1.00/1.00	1.00/0.002
	positive gain	1.00/1.00	1.00/1.00	1.00/1.00
	non-negative gain	1.00/1.00	1.00/1.00	1.00/<0.001

**A.3. Exact values for RQ3**

**Table 21.** Exact quantitative results corresponding to the visualization (bars) in figure 2.

fine-tune on →	card			coin			dice		
test on →	card	coin	dice	card	coin	dice	card	coin	dice
↓ model	train set(Acc)/test set(Acc)								
BERT	100/100	75/76	75/77	75/82	100/100	50/50	85/83	74/69	100/99
RoBERTa	100/100	75/75	75/75	75/72	100/97	50/42	75/73	75/73	100/96
DeBERTa	100/98	96/88	99/93	100/95	100/97	75/67	72/58	100/83	85/85
BigBird	99/90	54/56	71/67	81/59	100/100	100/97	65/63	51/50	97/91
